# Optimization of substrate formulation for *Hericium erinaceus* by replacing wood by straw and their effect on enzyme activities

**DOI:** 10.3389/fpls.2024.1436385

**Published:** 2024-12-13

**Authors:** Zhu Lu, Lingyun Liu, Ziming Ren, Shuang Hu, Yue Wang, Shujuan Ji, Xu Wang, Zhongwei Du, Yanni Liu, Yang Yang, Yanshen Yu

**Affiliations:** ^1^ Jilin Province Vegetable and Flower Research Institute, Changchun, China; ^2^ Environment and Plant Protection Institute, Chinese Academy of Tropical Agricultural Sciences, Haikou, China; ^3^ Engineering Research Center of Edible and Medicinal Fungi, Ministry of Education, Jilin Agricultural University, Changchun, China; ^4^ Jilin Sericulture Science Research Institute, Changchun, China; ^5^ Yanbian Academy of Agricultural Sciences, Yanbian, China

**Keywords:** *Hericium erinaceus*, substrate formulation, replacing wood by grass, simplex-lattice method, enzyme activity

## Abstract

**Introduction:**

China is rich in straw resources. The utilization of straw in the cultivation of edible fungi partially resolves the resource conflicts between mushroom cultivation and forest industry and also contributes to environmental protection.

**Methods:**

In this study, based on the technology of replacing wood by grass, the straw formula for mycelial culture of *Hericium erinaceus* was optimized with Simplex-lattice method commonly used in mixture design. By measuring the growth rate and the activity of lignocellulose degrading enzymes of mycelia in different formulations, and further combining with model optimization, the optimal formulation was screened and validated for mushroom cultivation.

**Results:**

In the experiments, different kinds of straw used as the main material showed interaction effects, further affecting the growth rate of mycelia and the activities of laccase, cellulase, and neutral xylanase. The screened optimal formula was composed of 16.3% rice straw, 59.7% cob, 20.0% wheat bran, 2.0% gypsum, 1.0% sucrose, and 1.0% calcium superphosphate. In the mushroom cultivation, 445.69 g of fresh mushroom were obtained and the biological efficiency reached 89.14%. The growth period of the first mushroom was shortened by 7-9 days. Some nutritional components of fruiting bodies, such as crude fats (6.10%), crude proteins (152.02 g/kg), K (19.71 g/kg), P (2.48 g/kg), and Se (6.06 g/kg), were significantly higher than those of the control formula.

**Discussion:**

These above indicators indicated that the screened formula could be applied in the high-yield and high-quality cultivation of *H. erinaceus*. Our study lays the foundation for expanding cultivation and strains improvement of *H. erinaceus*, and is conducive in promoting the rapid development of *H. erinaceus* industry.

## 1 Introduction

H. *erinaceus* is a precious edible and medicinal mushroom with delicious taste and high nutritional value (Friedman, 2015; Sangtitanu et al., 2020). Its fruiting bodies are rich in polysaccharide polypeptides, essential amino acids, and trace elements (Gong et al., 2004; Zhu et al., 2014). *H. erinaceus* has a variety of pharmacological properties, such as relieving gastric diseases (Yuan et al., 2021), anti-inflammatory (Yao et al., 2015; Tada et al., 2022), anti-cancer (Li et al., 2015; Liu et al., 2020), and liver protection (Cui et al., 2016; Jiang et al., 2016). The wide range of medicinal values of *H. erinaceus* has been increasingly concerned and its demand and cultivation scale have been increasing. As a typical wood-decay fungus, *H. erinaceus* utilizes wood as the main raw material. However, with the rapid development of the mushroom industry in recent years, the resource conflict between mushroom cultivation and forestry has become increasingly prominent, and the expansion of the cultivation industry of edible fungi are facing difficulties. Therefore, it is necessary to search for new raw materials for *H. erinaceus* cultivation with the reduced cost.

China is one of the countries with the richest straw resources in the world and the average annual output of straw in China reaches 700 million tons (Yang et al., 2023). Traditional straw treatment methods include open burning and landfilling, which caused waste and serious environmental pollution (He et al., 2016; Montero et al., 2016; He et al., 2018). Replacing wood by grass is a new technology developed in recent years in the edible fungus industry. With the new technology, herbaceous plants (mainly gramineous plants) and waste agricultural residues were used in mushrooms cultivation. This technology can resolve the resource conflicts between mushroom cultivation and forest industry, reduce the cultivation cost of edible fungi, utilize biological resources (Dedousi et al., 2024), decrease environmental pollution, and realize the sustainable development of edible fungus industry. At present, this technology has been widely applied in the cultivation of *Auricularia auricula* (Zhang et al., 2016), *Pleurotus pulmonarius* (Wang et al., 2022), *Pholiota microspora* (Meng et al., 2019), *Pleurotus eryngii* (Moonmoon et al., 2010), and *Lentinula edodes* (Pedri et al., 2015). In addition, Simplex-lattice method in mixing design has been applied by mushroom cultivators in the optimization of edible fungus straw cultivation formulas due to its advantages of less test points, simple statistical methods, and high fitting accuracy and has significantly increased mushroom production (Song et al., 2018; Wu et al., 2019a).

During the growth process of edible fungi, they decompose lignin, cellulose, and other biomacromolecules in the substrate. In the growth process, extracellular enzymes play an indispensable role and the activities of extracellular enzymes are related to physicochemical properties of culture media, species of edible fungi, and growth stage (Ball and Jackson, 1995; Kabel et al., 2017; Cesur et al., 2022). At present, the extracellular enzymes of edible fungi detected in production mainly include cellulase (Sun et al., 2021), xylanase (Agustinho et al., 2021), laccase, protease (Majumder et al., 2016) and SOD enzyme, which largely improve the utilization rate of the substrate and the growth rate of mycelium. The measured activities of these extracellular enzymes can be used to guide the high-yield cultivation of edible fungi.

In this study, we screened suitable cultivation substrates from domestic major crop straws and designed the substrate formulas with Simplex-lattice method. The relationships between mycelial growth rate and extracellular enzyme activities in different formulas were analyzed to determine suitable formulas for the cultivation of *H. erinaceus*. Our study lays the foundation for the large-scale cultivation of *H. erinaceus* with the technology of replacing wood by grass.

## 2 Materials and methods

### 2.1 Test strain

*H. erinaceus* 20190111, one strain with fast growth rate (4.92mm/d), high yield (biological efficiency 91.85%), and good quality screened by previous germplasm resource evaluation, was provided by the Center of Edible Fungus Research of Jilin Province Vegetable and Flower Research Institute, China.

### 2.2 Screening straw for the cultivation formula with wood replaced with grass

Based on the basic substrate formula (76.0% wood chips, 20.0% wheat bran, 2.0% gypsum, 1.0% sucrose, 1.0% superphosphate, and 62.0% water), all wood chips (76.0%) were completely replaced by corn straw, cob, rice straw, wheat straw, soybean straw, peanut straw and rapeseed straw, respectively. After the materials were completely mixed, spread in petri dishes (9 cm in diameter), sterilized, and then cooled for use. Round pieces (6 mm in diameter) of mycelia (cultured for 7-10 d on PDA medium at 25 °C, with darkness) were inoculated in the middle of petri dish, and the culture conditions (25 °C, dark) were invariable. When the mycelia were fully grown, draw “+” mark on the back of the petri dishes with the inoculation point as the center, and use the cross-hatch method to measure the colony diameter (Shrestha et al., 2006; Dedousi et al., 2024). Use SPSS software to calculate the daily growth rate of mycelia and perform analysis of variance, with 10 biological replicates per treatment.

### 2.3 Design and optimization of straw formulas with wood replaced with grass

The Simplex-lattice method in the software Design-Expert 8.0.6.1, which limit the upper and lower boundaries of various components in mixture design (Ghorbani et al., 2021; Jeswani et al., 2021; Ghislain et al., 2022), was used to design and optimize straw formulas for the growth of *H. erinaceus* mycelia. Based on the preliminary screening results, suitable kinds of straw were selected as the main ingredient (X) to replace wood chips, and the replacement ratio of each straw were set as the level of the investigation factor. All replacement ratios need to meet X_n_ ≥ 0, and X_1_+X_2_+…+X_n_=1. After obtaining the replacement ratio of each straw, convert it according to the addition ratio of wood chips (76%) in the formula, and finally obtain the actual addition ratio of each straw. The mycelial growth rate and the activities of laccase (Ren et al., 2023), cellulase (Zhao et al., 2020), and neutral xylanase (Agustinho et al., 2021) of each formula were further determined and the correlation analysis was performed to establish quadratic regression models for each main material. The effects of the changes and interactions of the components in the ratios of the substrates on mycelial growth were analyzed. Further validate the authenticity of the model and optimize its parameters to maximize the response value, and finally obtain the optimal formula.

### 2.4 Verification test

The optimized formula and control formula were simultaneously verified in mycelial growth test and cultivation test at the Jilin Province Vegetable and Flower Research Institute. The growth conditions (25 °C, dark) in mycelial growth test were consistent with those in straw screening (section 2.2), and the mycelial growth rates were measured using cross-hatch method, and the activities of laccase, cellulase, and neutral xylanase were detected with microcalorimetry enzyme activity assay kits (COMIN, Suzhou, China). The cultivation conditions for the cultivation test were set according to Zhu et al. (2019), and make appropriate modifications. During the mycelial stage, the temperature was controlled at 25 °C, the relative humidity of the air was 60%, and avoid light. After 30 days, the cultivation temperature should be adjusted to 17-22 °C for post ripening (10 d), while keeping other conditions unchanged. Then transferred the mushroom bags to the mushroom room for fruiting, and provided weak scattered light. The temperature should be controlled at 15-18 °C, the air humidity was 90-95%, and the CO2 concentration was 0.03% (no special treatment was required for bud pressing). The cultivation period from inoculation to mushroom emergence was about 50-60 days, and the agronomic traits such as primordia formation time, fresh mass, biological efficiency, color, shape, firmness, fungal spines, and anti-bacteria capacity were recorded. In the above tests, 20 replicates were arranged. Furthermore, the contents of the nutrients in the fruiting bodies of *H. erinaceus* cultivated in two formulas were detected, including crude fat (Soxhlet method), crude protein (Kjeldahl method), crude fiber (acid-base hydrolysis method), ash (burning method, GB 5009.4-2016), and polysaccharides (phenol sulfuric acid method). Meanwhile, the trace elements (K, Ca, Fe, Zn, Mn, Cu, P, and Se) were also detected using the ICP-MS method in three replicates. The above results were comprehensively analyzed to verify the feasibility of the optimized formula. The results of mycelial growth rate, enzyme activity, nutrient contents and trace elements were analyzed by SPSS software and plotted using Origin software. The Design Expert 8.0.6.1 software was used for formula design, correlation analysis and contour drawing.

### 2.5 Statistical analysis

The variance analysis of mycelial growth rate, enzyme activity, nutrient contents and trace elements were conducted using SPSS software, and the corresponding figures were drawn using Origin software. The correlation analysis and linear regression analysis between mycelial growth rate/enzyme activity and various straws, as well as the corresponding contour drawing, were conducted in the Design Expert 8.0.6.1 software.

## 3 Results

### 3.1 Straw screening test

**Figure 1:**
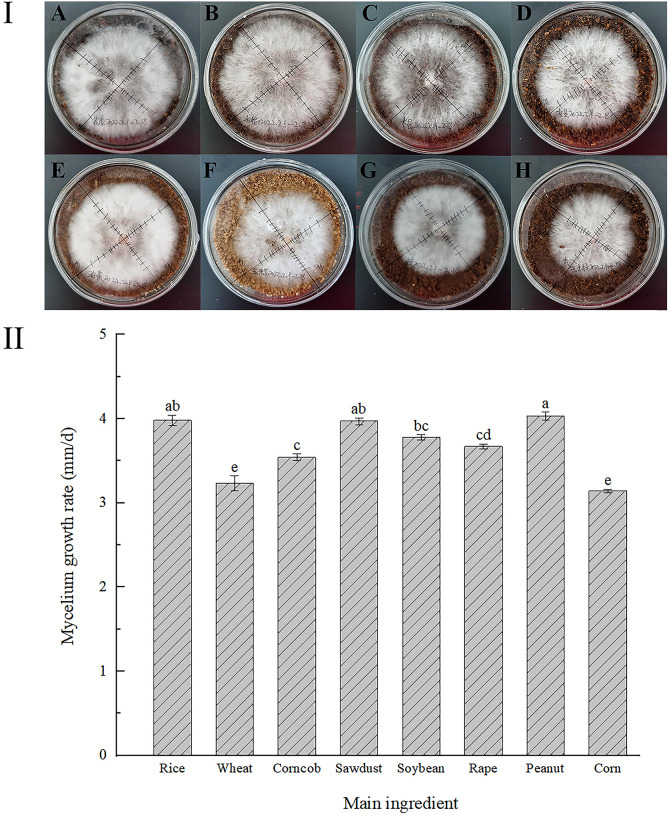
Growth states of *H. erinaceus* mycelium on different straw medium plates. (**I-A)**: Peanut straw; **(I-B)**: Rice straw; **(I-C)**: Wood chips; **(I-D)**: Soybean straw; **(I-E)**: Rapeseed straw; **(I-F)**: Cob; **(I-G)**: Wheat straw; **(I-H)**: Corn straw; **II**: Mycelial growth rate. Significance, different lowercase letters represent significant differences, and the same letter represents insignificant differences).

As shown in **Figure 1**, the mycelia growth rate was the fastest on peanut straw (4.03 mm/d) and rice straw (3.98 mm/d), which were not significantly different from that on wood chips. Next were soybean straw (3.78 mm/d), rapeseed straw (3.67 mm/d), and corn cob (3.54mm/d), with the slowest growth rate on wheat straw and corn straw. Therefore, a total of five kinds of straws (rice, soybean, peanut, rapeseed straw, and cob) were initially selected as the main substrates for mycelial culture of *H. erinaceus.*

### 3.2 Mixture formula and measurement results

A total of 21 formulas were designed by constraining the proportion of each ingredient in the main material with the design software (**Table 1**). The mycelial growth rate in formulas 1, 6, 12, and 17 was significantly faster than that in the control formula (CK). The laccase activity in formula 2 was the highest and the laccase activity in most formulas was higher than that in CK. The cellulase activity in formula 4 was the highest and much higher than that in CK. The neutral xylanase activity in Formula 8 was the highest and much higher than that in other formulas and CK. In short, different straw formulas had significant effects on the mycelial growth rate and enzyme activities of *H. erinaceus*, and some straws were more conducive to the production of extracellular enzymes in *H. erinaceus* mycelia than wood chips.

**Table 1:** Formula design and measurement results of culture materials.

Formula	Straw (%)					Average growth rate(cm/d)	Laccase activity (nmol/min/g)	Cellulase activity (μg/min/g)	Neutral xylanase (nmol/min/g)
	X_1_ Rice straw	X_2_ Soybean straw	X_3_ Cob straw	X_4_ Peanut straw	X_5_ rapeseed straw				
1	100	0	0	0	0	4.52 ± 0.01^a^	77.85 ± 2.38^gh^	749.47 ± 7.92^de^	3658.81 ± 111.76^gh^
2	0	100	0	0	0	4.19 ± 0.07^fg^	346.44 ± 79.87^a^	808.19 ± 1.22^bcd^	3607.70 ± 88.17^gh^
3	0	0	100	0	0	4.17 ± 0.06^fg^	189.93 ± 5.81^c^	778.12 ± 1.98^cde^	5563.85 ± 434.66^de^
4	0	0	0	100	0	4.22 ± 0.1^efg^	170.64 ± 8.52^cd^	1028.87 ± 146.05^a^	4046.91 ± 112.39^gh^
5	0	0	0	0	100	3.96 ± 0.13^h^	135.35 ± 10.22^ef^	809.69 ± 0.92^bcd^	6672.49 ± 361.39^c^
6	50	50	0	0	0	4.54 ± 0.01^a^	145.53 ± 11.37^de^	832.29 ± 16.71^bcd^	7673.40 ± 583.29^b^
7	50	0	50	0	0	4.27 ± 0.11^def^	43.70 ± 5.63^i^	861.90 ± 67.67^bc^	6768.57 ± 573.17^c^
8	50	0	0	50	0	4.21 ± 0.02^efg^	136.98 ± 11.47^def^	797.00 ± 61.21^bcd^	8859.79 ± 377.91^a^
9	50	0	0	0	50	3.8 ± 0.08^i^	143.02 ± 4.14^de^	572.05 ± 53.61^f^	4790.94 ± 388.66^f^
10	0	50	50	0	0	3.96 ± 0.09^h^	156.45 ± 7.80^de^	871.98 ± 58.28^b^	5014.28 ± 216.97^ef^
11	0	50	0	50	0	4.1 ± 0.01^g^	99.02 ± 4.20^gh^	833.88 ± 26.76^bcd^	1302.72 ± 77.69^j^
12	0	50	0	0	50	4.54 ± 0.02^a^	284.62 ± 8.65^b^	887.90 ± 6.50^b^	1884.65 ± 107.93^i^
13	0	0	50	50	0	4.39 ± 0.02b^cd^	256.62 ± 10.33^b^	885.74 ± 9.18^b^	6023.20 ± 215.44^de^
14	0	0	50	0	50	4.18 ± 0.03^fg^	146.69 ± 1.00^de^	869.44 ± 43.69^bc^	4908.71 ± 177.69^f^
15	0	0	0	50	50	4.32 ± 0.03^de^	162.74 ± 7.32^cde^	699.29 ± 19.55^e^	3444.51 ± 156.56^h^
16	60	10	10	10	10	4.21 ± 0.09^efg^	74.94 ± 4.48^ghi^	848.21 ± 35.94^bc^	6067.39 ± 375.66^de^
17	10	60	10	10	10	4.46 ± 0.07^ab^	107.06 ± 3.15^fg^	842.51 ± 33.22^bc^	1994.17 ± 12.30^i^
18	10	10	60	10	10	4.34 ± 0.04^cde^	139.88 ± 2.49^de^	858.58 ± 22.85^bc^	5293.96 ± 360.79^ef^
19	10	10	10	60	10	4.17 ± 0.09^fg^	93.17 ± 3.19^gh^	857.60 ± 30.43^bc^	5050.34 ± 502.71^ef^
20	10	10	10	10	60	4.21 ± 0.08^efg^	99.61 ± 4.30^gh^	841.19 ± 14.86^bc^	6064.74 ± 499.49^de^
21	20	20	20	20	20	4.27 ± 0.08^def^	88.56 ± 6.11^gh^	800.50 ± 14.66^bcd^	5238.31 ± 325.40^ef^
Ck	Wood chips 100					4.44 ± 0.04^abc^	68.29 ± 1.38^hi^	816.05 ± 61.58^bcd^	2357.59 ± 165.70^i^

Different lowercase letters in the table represent significant differences, whereas the same letter represents insignificant differences.

### 3.3 Correlation analysis

#### 3.3.1 Correlations between mycelial growth rate and various straws of *H. erinaceus*

The regression equation between mycelial growth rate and each main material is expressed as: Y=4.52X_1_ + 4.19X_2_ + 4.17X_3_ + 4.22X_4_ + 3.96X_5_ + 0.74X_1_X_2_ - 0.30X_1_X_3_ - 0.65X_1_X_4_ - 1.76X_1_X_5_ - 0.86X_2_X_3_ - 0.41X_2_X_4_ + 1.88X_2_X_5_ + 0.76X_3_X_4_ + 0.44X_3_X_5_ + 0.90X_4_X_5_ + 103.37X_1_^2^X_2_X_3_ - 280.97X_1_^2^X_2_X_4_ + 67.85X_1_^2^X_2_X_5_ + 78.16X_1_^2^X_3_X_4_ + 64.99X_1_X_2_^2^X_3_, correlation coefficient of R^2^ = 0.9178. (1)

**Figure 2:**
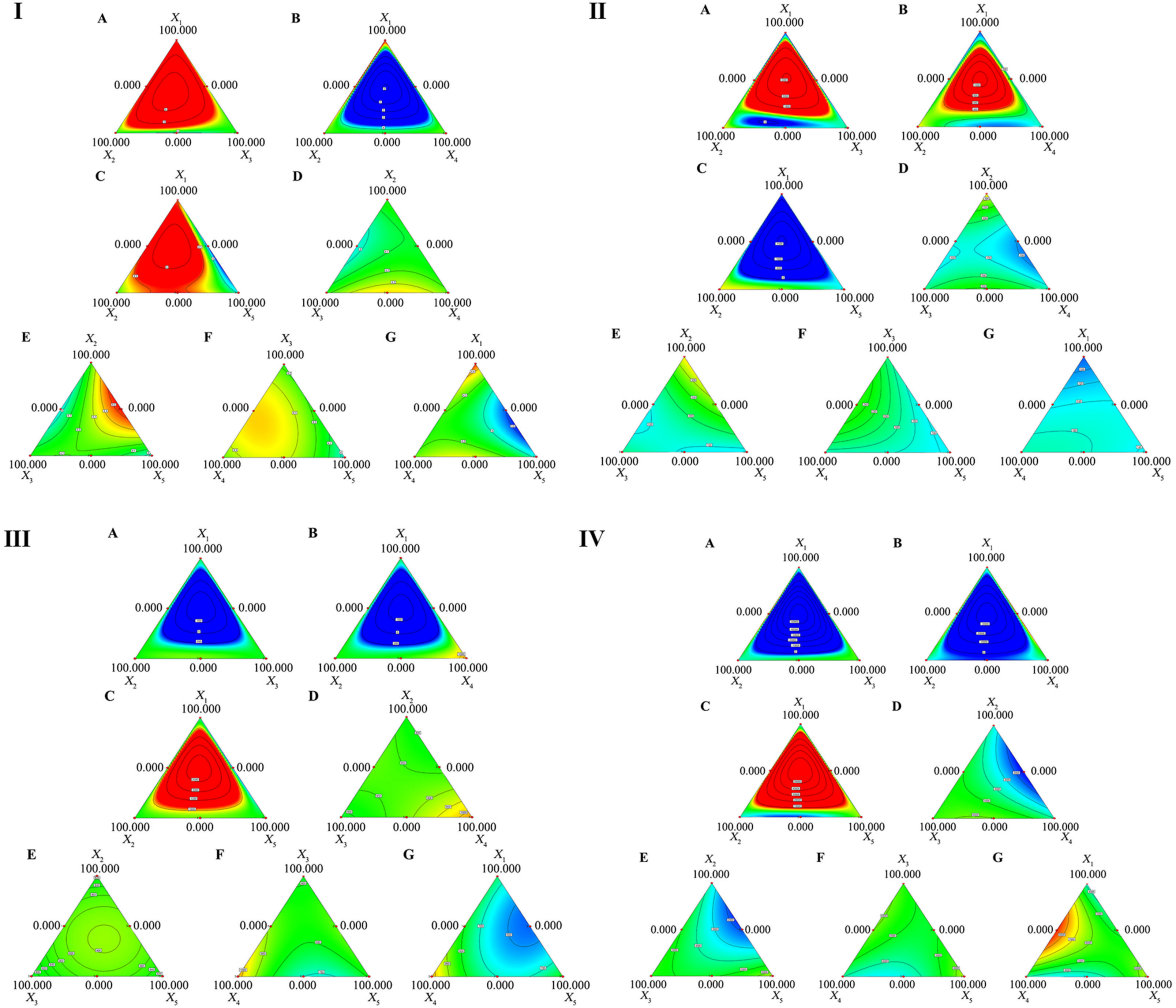
Contour map. I: Contour Figure **(I-A)** shows the effect of the interaction between various main ingredients on mycelial growth rate: the interaction among rice straw, soybean straw, and cob; **(I-B)**: The interaction between rice straw, soybean straw, and peanut straw; **(I-C)**: The interaction between rice straw, peanut straw, and rapeseed straw; **(I-D)**: The interaction between soybean straw, cob, and peanut straw; **(I-E)**: The interaction between soybean straw, cob straw, and rapeseed straw; **(I-F)**: The interaction between cob, peanut straw, and rapeseed straw; **(I-G)**: The interaction between rice straw, peanut straw, and rapeseed straw. **II**: Contour Figure **(II-A)**: The interaction between rice straw, soybean straw, and cob; **(II-B)**: The interaction between rice straw, soybean straw, and peanut straw; **(II-C)**: The interaction between rice straw, peanut straw, and rapeseed straw; **(II-D)**: Interaction between soybean straw, cob, and peanut straw; **(II-E)**: The interaction between soybean straw, cob straw, and rapeseed straw; **(II-F)**: The interaction between cob, peanut straw, and rapeseed straw; **(II-G)**: The interaction between rice straw, peanut straw, and rapeseed straw. **III**: Contour Figure **(III-A)**: The interaction between rice straw, soybean straw, and cob; **(III-B)**: Interaction between rice straw, soybean straw, and peanut straw; **(III-C)**: Interaction among rice straw, peanut straw, and rapeseed straw; **(III-D)**: Interaction among soybean straw, cob, and peanut straw; **(III-E)**: Interaction among soybean straw, cob straw, and rapeseed straw; **(III-F)**: Interaction among cob, peanut straw, and rapeseed straw; **(III-G)**: The interaction between rice straw, peanut straw, and rapeseed straw. **IV**: Contour **(IV-A)**: The interaction between rice straw, soybean straw, and cob; **(IV-B)**: The interaction between rice straw, soybean straw, and peanut straw; **(IV-C)**: The interaction between rice straw, peanut straw, and rapeseed straw; **(IV-D)**: Interaction between soybean straw, cob, and peanut straw; **(IV-E)**: The interaction between soybean straw, cob straw, and rapeseed straw; **(IV-F)**: The interaction between cob, peanut straw, and rapeseed straw; **(IV-G)**: The interaction between rice straw, peanut straw, and rapeseed straw. The closer the color of the contour map is to red, the higher the numerical value and the contribution rate; the closer it is to blue, the lower the numerical value and the contribution rate).

Based on the variance analysis of the quadratic multiple regression model for fitting mycelial growth rate (**Table 2**), the *P*-values of the linear mixed model and the quadratic regression model were both less than 0.0001, suggesting both models fitted the relationship between the main ingredients and mycelial growth rate well, and the data could be used in the subsequent analysis. From the regression coefficients (K value) of the regression equation, it can be inferred that the effects degree of various straws on mycelial growth rate was in the following order: rice straw (K_X1_ = 4.52) > peanut straw (K_X4_ = 4.22) > soybean straw (K_X2_ = 4.19) > cob (K_X3_ = 4.17) > rapeseed straw (K_X5_ = 3.96). According to the analysis of variance (**Table 2**), the interaction terms of X_1_X_2_, X_1_X_4_, X_1_X_5_, X_2_X_3_, X_2_X_5_, X_3_X_4_, X_4_X_5_ and X_1_X_2_^2^X_3_ were extremely significant (p ≤ 0.01), indicating that their interaction can significantly affect the mycelial growth of *H. erinaceus*. Based on the regression equation and contour plot analysis (**Figure 2I**): for X_1_X_2_(k=0.74, red), X_1_X_4_(k=-0.65, blue) and X_1_X_5_(k=-1.76, blue), the effect of X_1_ on mycelia growth was associated with the addition of X_2_, X_4_, and X_5_ in the formula; for X_2_X_3_ (k=-0.86, blue), X_2_X_5_(k=1.88, red) and X_1_X_2_^2^X_3_(k=64.99), the effect of X_2_ on mycelia growth was associated with the addition of X_3_, X_5_ and X_1_-X_3_ (simultaneously add X_1_ and X_3_) in the formula; for X_3_X_4_(k=0.76, red), adding X_4_ to the formula can enhance the effect of X_3_ on mycelial growth rate, indicating that their interaction was beneficial for mycelia growth; for X_4_, the interaction effect (k=0.90, red) of adding X_5_ to the formula was beneficial for improving the mycelial growth rate.

**Table 2:** ANOVA for the fitted quadratic polynomial model of mycelium growth rate.

Sources	Sun of squares	*df*	Mean square	*F*	*P*
Model	2.20	19	0.12	25.26	*<0.0001*
Linear mixed model	0.29	4	0.073	15.92	*<0.0001*
X_1_X_2_	0.069	1	0.069	15.10	0.0003
X_1_X_3_	0.011	1	0.011	2.5	0.1215
X_1_X_4_	0.053	1	0.053	11.49	0.0015
X_1_X_5_	0.39	1	0.39	84.73	*<0.0001*
X_2_X_3_	0.093	1	0.093	20.29	*<0.0001*
X_2_X_4_	0.021	1	0.021	4.57	0.0383
X_2_X_5_	0.44	1	0.44	96.14	*<0.0001*
X_3_X_4_	0.072	1	0.072	15.65	0.0003
X_3_X_5_	0.025	1	0.025	5.38	0.0252
X_4_X_5_	0.10	1	0.10	21.97	*<0.0001*
X_1_^2^X_2_X_3_	3.347E-003	1	3.347E-003	0.73	0.3976
X_1_^2^X_2_X_4_	0.032	1	0.032	6.96	0.0115
X_1_^2^X_2_X_5_	3.004E-003	1	3.004E-003	0.66	0.4227
X_1_^2^X_3_X_4_	1.687E-003	1	1.687E-003	0.37	0.5473
X_1_X_2_^2^X_3_	0.041	1	0.041	8.96	0.0046
Residual	0.20	1	0.20		
Lack of fit	3.19E-003	1	3.19E-003	0.69	0.4104
Pure error	0.19	42	4.617E-003		
Cor total	2.40	62			

*F*, Evaluate whether the influence of inter group factors is significant, and larger *F*-value represents more significant difference between groups compared to within group differences. *P*, Evaluate whether the impact is statistically significant, and smaller *P*-value indicating the higher statistical significance of the result.

#### 3.3.2 Correlation of laccase activity and various straws of *H. erinaceus*

The regression equation between laccase activity and each main material is expressed as: Y = 76.91X_1_ + 345.50X_2_ + 188.99X_3_ + 169.70X_4_ + 134.41X_5_ - 264.85X_1_X_2_ - 359.14X_1_X_3_ + 52.54X_1_X_4_ + 147.29X_1_X_5_ - 445.31X_2_X_3_ - 636.46X_2_X_4_ + 176.52X_2_X_5_ + 306.94X_3_X_4_ - 62.18X_3_X_5_ + 40.58X_4_X_5_ + 2.134E + 0.05X_1_^2^X_2_X_3_ + 60765.33X_1_^2^X_2_X_4_ - 1.082E + 005X_1_^2^X_2_X_5_ - 1.633E + 005X_1_^2^X_3_X_4_ - 46157.78X_1_X_2_^2^X_3_, correlation coefficient of R^2^ = 0.9230. (2)

Based on the variance analysis of the quadratic multiple regression model for laccase activity (**Table 3**), the *P*-values of the linear mixed model and the quadratic regression model were both less than 0.0001, suggesting both models fitted the relationship between the main ingredients and laccase activity well, and the data could be used in the subsequent analysis. From the regression coefficients (K value) of the regression equation, it can be inferred that the effects degree of five kinds of straw on laccase activity ranked in the following decreasing order: soybean straw (K_X2_ = 345.50) > cob (K_X3_ = 188.99) > peanut straw (K_X4_ = 169.70) > rapeseed straw (K_X5_ = 134.41) > rice straw (K_X1_ = 76.91). According to the analysis of variance (**Table 3**), the *p*-values corresponding to the interaction terms of X_1_X_2_, X_1_X_3_, X_1_X_5_, X_2_X_3_, X_2_X_4_, X_2_X_5_, X_3_X_4_, X_1_^2^X_2_X_3_, X_1_^2^X_2_X_5_, X_1_^2^X_3_X_4_, and X_1_X_2_^2^X_3_ were all less than 0.001, indicating that their interaction can significantly affect the laccase activity of *H. erinaceus*. Based on the regression equation and contour plot analysis (**Figure 2II**): for X_1_X_2_ (k=-264.5, blue), X_1_X_3_ (k=-359.14, blue), X_1_X_5_ (k=147.25, red), X_1_^2^X_2_X_3_ (k=0.05, red), X_1_^2^X_2_X_5_ (k=0.05, red) and X_1_^2^X_3_X_4_(k=0.05, red), the effect of X_1_ on laccase activity was associated with the amount of X_2_, X_3_, X_5_, X_2_-X_3_ and X_2_-X_5_ in the formula; for X_2_X_3_ (k=-445.31, blue), X_2_X_4_(k=-636.46, blue), X_2_X_5_(k=176.52, red) and X_1_X_2_^2^X_3_(k=-46157.78), the effect of X_2_ on laccase activity was associated with the addition of X_3_, X_4_, X_5_ and X_1_-X_3_ in the formula; for X_3_X_4_(k=306.94, red), adding X_4_ to the formula can enhance the effect of X_3_ on laccase activity, indicating that their interaction was beneficial for lignin degradation.

**Table 3:** ANOVA for the fitted quadratic polynomial model of laccase activity.

Source	Sun of squares	*df*	Mean square	*F*	*P*
Model	3.19E+05	19	16805.74	45.32	*< 0.0001*
Linear mixed model	1.16E+05	4	29011.96	78.24	*< 0.0001*
X_1_X_2_	8770.3	1	8770.3	23.65	*< 0.0001*
X_1_X_3_	16126.91	1	16126.91	43.49	*< 0.0001*
X_1_X_4_	345.11	1	345.11	0.93	0.3401
X_1_X_5_	2712.52	1	2712.52	7.32	0.0098
X_2_X_3_	24793.68	1	24793.68	66.87	*< 0.0001*
X_2_X_4_	50647.09	1	50647.09	136.59	*< 0.0001*
X_2_X_5_	3896.09	1	3896.09	10.51	0.0023
X_3_X_4_	11779.81	1	11779.81	31.77	*< 0.0001*
X_3_X_5_	483.45	1	483.45	1.3	0.2598
X_4_X_5_	205.87	1	205.87	0.56	0.4602
X_1_^2^X_2_X_3_	14262.73	1	14262.73	38.47	*< 0.0001*
X_1_^2^X_2_X_4_	1493.37	1	1493.37	4.03	0.0511
X_1_^2^X_2_X_5_	7637.26	1	7637.26	20.6	*< 0.0001*
X_1_^2^X_3_X_4_	7361.1	1	7361.1	19.85	*< 0.0001*
X_1_X_2_^2^X_3_	20709.83		20709.83	55.85	*< 0.0001*
Residual	15944.16	43	370.79		
Lack of fit	1299.64	1	1299.64	3.73	0.0603
Pure error	14644.52	42	348.68		
Cor total	3.35E+05	62			

#### 3.3.3 Correlations between cellulase activity and various straws of *H. erinaceus*

The regression equation between cellulase activity and each main material is expressed as: Y=751.86X_1_ + 810.57X_2_ + 780.51X_3_ + 1031.26X_4_ + 812.07X_5_ + 209.77X_1_X_2_ + 388.33X_1_X_3_ - 372.77X_1_X_4_ - 834.22X_1_X_5_ + 311.21X_2_X_3_ - 342.71X_2_X_4_ + 311.75X_2_X_5_ - 75.14X_3_X_4_ + 298.05X_3_X_5_ - 884.07X_4_X_5_ -1.106E + 005X_12_X_2_X_3_ - 96289.73X_1_^2^X_2_X_4_ + 1.307E + 005X_1_^2^X_2_X_5_ + 97272.77X_1_^2^X_3_X_4_ + 7313.02X_1_X_2_^2^X_3_, correlation coefficient of R^2^ = 0.8195. (3)

Based on the variance analysis of the quadratic multiple regression model for cellulase activity (**Table 4**), the *P*-values of the linear mixed model and the quadratic regression model were both less than 0.0001, suggesting both models fitted the relationship between the main ingredients and cellulase activity well, and the data could be used in the subsequent analysis. From the regression coefficients (K value) of the regression equation, it can be inferred that the effects degree of five kinds of straw on cellulase activity ranked in the following decreasing order: peanut straw (K_X4_ = 1031.26) > rapeseed straw (K_X5_ = 812.07) > soybean straw (K_X2_ = 810.57) > cob (K_X3_ = 780.51) > rice straw (K_X1_ = 751.86). As shown in **Table 4**, The *p*-values corresponding to the interaction terms of X_1_X_3_, X_1_X_4_, X_1_X_5_ and X_4_X_5_ were all less than 0.01, indicating that their interaction can significantly affected the cellulase activity of *H. erinaceus*. Based on the regression equation and contour plot analysis (**Figure 2III**), for X_1_X_3_(k=388.33, red), X_1_X_4_(k=-372.77, blue) and X_1_X_5_(k=-834.22, blue), the effect of X_1_ on cellulase activity was associated with the amount of X_3_, X_4_ and X_5_ in the formula; for X_4_, the interaction effect (k=-884.07, blue) of adding X_5_ to the formula was not conducive to cellulose degradation.

**Table 4:** ANOVA for fitted quadratic polynomial model of cellulase activity.

Source	Sun of squares	*df*	Mean square	*F*	*P*
Model	4.35E+05	19	22891.07	10.28	*< 0.0001*
Linear mixed model	1.39E+05	4	34649.88	15.56	*< 0.0001*
X_1_X_2_	5501.69	1	5501.69	2.47	0.1234
X_1_X_3_	18855.16	1	18855.16	8.47	0.0057
X_1_X_4_	17373.61	1	17373.61	7.8	0.0078
X_1_X_5_	87011.88	1	87011.88	39.07	*< 0.0001*
X_2_X_3_	12109.31	1	12109.31	5.44	0.0245
X_2_X_4_	14684.56	1	14684.56	6.59	0.0138
X_2_X_5_	12151.37	1	12151.37	5.46	0.0242
X_3_X_4_	705.91	1	705.91	0.32	0.5764
X_3_X_5_	11106.83	1	11106.83	4.99	0.0308
X_4_X_5_	97720.89	1	97720.89	43.87	*< 0.0001*
X_1_^2^X_2_X_3_	3831.75	1	3831.75	1.72	0.1966
X_1_^2^X_2_X_4_	3749.86	1	3749.86	1.68	0.2014
X_1_^2^X_2_X_5_	11148.51	1	11148.51	5.01	0.0305
X_1_^2^X_3_X_4_	2612.56	1	2612.56	1.17	0.2848
X_1_X_2_^2^X_3_	519.85	1	519.85	0.23	0.6315
Residual	95772.73	43	2227.27		
Lack of fit	8383.73	1	8383.73	4.03	0.0512
Pure error	87389.01	42	2080.69		
Cor total	5.31E+05	62			

#### 3.3.4 Correlations between neutral xylanase activity and various straws of *H. erinaceus*

The regression equation between neutral xylanase activity and each main material is expressed as: Y=3646.14X_1_ + 3595.03X_2_ + 5551.18X_3_ + 4034.24X_4_ + 6659.82X_5_ + 16182.29X_1_X_2_ + 8650.69X_1_X_3_ + 20049.46X_1_X_4_ - 1477.11X_1_X_5_ + 1735.73X_2_X_3_ - 10076.61X_2_X_4_ - 13000.07X_2_X_5_ + 4892.99X_3_X_4_ - 4816.13X_3_X_5_ - 7639.03X_4_X_5_ - 3.971E + 006X_1_^2^X_2_X_3_ - 2.700E + 006X_1_^2^X_2_X_4_ + 3.354E + 006X_1_^2^X_2_X_5_ + 3.235E + 006X_1_^2^X_3_X_4_ - 83254.70X_1_X_2_^2^X_3_, correlation coefficient of R^2^ = 0.9777. (4)

Based on the variance analysis of the quadratic multiple regression model for neutral xylanase activity (**Table 5**), the *P*-values of the linear mixed model and the quadratic regression model were both less than 0.0001, suggesting both models fitted the relationship between the main ingredients and neutral xylanase activity well, and the data could be used in the subsequent analysis. From the regression coefficients (K value) of the regression equation, it can be inferred that the effects of five kinds of straw on neutral xylanase activity ranked in the following decreasing order: rapeseed straw (KX_5_ = 6659.82) > cob (KX_3_ = 5551.18) > peanut straw (KX_4_ = 4034.24) > rice straw (KX_1_ = 3646.14) > soybean straw (KX_2_ = 3595.03). As shown in **Table 5**, except for X_1_X_5_, X_2_X_3_, and X_1_X_2_^2^X_3_, the *p*-values of other f interaction terms were less than 0.01, indicating that other interaction terms all can significantly affect the neutral xylanase activity of *H. erinaceus*. Based on the regression equation and contour plot analysis (**Figure 2IV**), for X_1_X_2_(k=16182.29, red), X_1_X_3_(k=8650.69, red), X_1_X_4_(k=20049.46, red), X_1_^2^X_2_X_3_ (k=0.06, red), X_1_^2^X_2_X_4_ (k=0.06, red), X_1_^2^X_2_X_5_ (k=0.06, red) and X_1_^2^X_3_X_4_(k=0.06, red), adding X_2_, X_3_, X_4_, X_2_-X_3_, X_2_-X_4_, X_2_-X_5_ or X_3_-X_4_ to the formula can enhance the effect of X_1_ on neutral xylanase activity, indicating that their interaction was beneficial for the hemicellulose degradation; for X_2_, the interaction effect of adding X_4_ (k=-10076.61, blue) or X_5_ (k=-13000.07, blue) to the formula was not conducive to hemicellulose degradation; for X_3_X_4_ (k=4892.99, red) and X_3_X_5_ (k=-4816.13, blue), the effect of X_3_ on neutral xylanase activity was associated with the amount of X_4_ and X_5_ in the formula; for X_4_, the interaction effect (k=-7639.03, blue) of adding X_5_ to the formula was not conducive to hemicellulose degradation.

**Table 5:** ANOVA for the fitted quadratic polynomial model of neutral xylanase activity.

Source	Sun of squares	*df*	Mean square	*F*	*P*
Model	2.13E+08	19	1.12E+07	99.29	*< 0.0001*
Linear mixed model	6.07E+07	4	1.52E+07	134.06	*< 0.0001*
X_1_X_3_	9.36E+06	1	9.36E+06	82.7	*< 0.0001*
X_1_X_4_	5.03E+07	1	5.03E+07	444.23	*< 0.0001*
X_1_X_5_	2.73E+05	1	2.73E+05	2.41	0.1278
X_2_X_3_	3.77E+05	1	3.77E+05	3.33	0.075
X_2_X_4_	1.27E+07	1	1.27E+07	112.21	*< 0.0001*
X_2_X_5_	2.11E+07	1	2.11E+07	186.77	*< 0.0001*
X_3_X_4_	2.99E+06	1	2.99E+06	26.46	*< 0.0001*
X_3_X_5_	2.90E+06	1	2.90E+06	25.63	*< 0.0001*
X_4_X_5_	7.30E+06	1	7.30E+06	64.49	*< 0.0001*
X_1_^2^X_2_X_3_	4.94E+06	1	4.94E+06	43.64	*< 0.0001*
X_1_^2^X_2_X_4_	2.95E+06	1	2.95E+06	26.07	*< 0.0001*
X_1_^2^X_2_X_5_	7.34E+06	1	7.34E+06	64.85	*< 0.0001*
X_1_^2^X_3_X_4_	2.89E+06	1	2.89E+06	25.54	*< 0.0001*
X_1_X_2_^2^X_3_	67375.87	1	67375.87	0.6	0.4445
Residual	4.87E+06	43	1.13E+05		
Lack of fit	2.36E+05	1	2.36E+05	2.14	0.1507
Pure error	4.63E+06	42	1.10E+05		
Cor total	2.18E+08	62			

### 3.4 Formula optimization and validation test

Based on the above regression equations, the expected response values of the evaluation indices were analyzed and set and then the formula with wood replaced by grass for the mycelial growth of *H. erinaceus* was optimized as follows: 16.3% rice straw, 59.7% cob, 20.0% wheat bran, 2.0% gypsum, 1.0% sucrose, and 1.0% calcium superphosphate. The validation results of plate test and mushroom production (**Table 6**) showed that mycelial growth rate, laccase activity, cellulase activity, and neutral xylanase activity of the optimized formula were better than that of control formula. The differences in agronomic traits or fruiting body morphology between the optimized formula and the control formula were not significant, but the differences in the contents of nutrients in fruiting bodies were significant. The contents of crude fats, crude proteins, K, P, and Se in the optimized formula were significantly higher than those in the control, but the contents of crude fibers, crude polysaccharides, Ca, Fe, Zn, and Cu in the optimized formula were lower. The results indicated that the optimized formula could replace the conventional wood chip formula for the cultivation of *H. erinaceus*.

**Table 6:** Comparison between optimized formula and control formula.

Categories	Test items	VF	CK
Mycelia stage	Mycelia growth rate (mm/d)	4.55 ± 032	4.43 ± 0.11
	Laccase activity (U/g)	80.67 ± 1.76	68.29 ± 2.02
	Cellulase activity (U/g)	858.98 ± 4.19	816.05 ± 8.25
	Neutral xylanase activity (U/g)	6075.54 ± 78.45	2357.59 ± 61.12
Mushroom emergence period	Primordium formation time (d)	7	8
	Average fresh weight (g)	445.69 ± 5.49	462.45 ± 3.87
	The fertility period of the first mushroom crop	50-53	57-59
	Average biological conversion rate (%)	89.14 ± 1.23	92.49 ± 3.46
	Fruiting body color	white	white
	Fruiting body shape	Round, monkey head-shaped	Round, monkey head-shaped
	Fungal spines	Long	Long
	Firmness of fruiting body	Tight and compact	Tight and compact
	Disease resistance	Strong	Strong
Ingredients of fruiting body	Crude fats (%) DW	6.10 ± 0.11	5.28 ± 0.12
	Crude proteins (g/kg) DW	152.02 ± 0.39	82.03 ± 0.59
	Crude fiber (%) DW	15.11 ± 0.13	17.00 ± 0.39
	Ash content (%) DW	7.78 ± 0.14	7.00 ± 0.08
	Crude polysaccharides (mg/g) DW	343.1 ± 0.55	435.13 ± 3.15
	Water content (%)	65.72 ± 0.07	64.26 ± 0.11
	K (g/kg) DW	19.71 ± 0.28	10.03 ± 0.09
	Ca (mg/kg) DW	240.63 ± 10.34	340.63 ± 10.34
	Fe (mg/kg) DW	88.04 ± 1.13	111.21 ± 2.20
	Zn (mg/kg) DW	10.45 ± 0.22	28.53 ± 0.85
	Mn (mg/kg) DW	4.37 ± 0.18	4.87 ± 0.06
	Cu (mg/kg) DW	8.10 ± 0.06	10.10 ± 0.34
	P (g/kg) DW	2.48 ± 0.15	1.53 ± 0.12
	Se (ug/kg) DW	6.06 ± 4.42	2.08 ± 0.27

## 4 Discussion

The cultivation substrate for edible fungus was usually composed of main material, auxiliary materials and water, providing carbon source, nitrogen source, and trace elements for the growth and development of mycelia and fruiting bodies. It was one of the three elements (strains, cultivation substrate, and cultivation technology) in the production of edible fungi. An appropriate cultivation formula can improve the growth state of edible fungi and reduce the cultivation cost. In recent years, the technology of replacing wood by grass has been used to optimize the cultivation formula of edible fungi. The technology resolved the resource conflicts between mushroom cultivation and forest industry and reduced the production cost. *H. erinaceus* is an important edible and medicinal mushroom and its cultivation area gradually increases. Therefore, the optimization of cultivation formula of *H. erinaceus* is important. In this study, corn straw, cob, rice straw, wheat straw, soybean straw, peanut straw, and rapeseed straw were collected in China and used in the optimization of mycelial growth formula of *H. erinaceus*, in order to obtain a high-yield and high-quality straw cultivation formula for the sustainable development of *H. erinaceus* industry.

With Simplex-lattice method, the quantitative relationships between matrix ratios and evaluation indexes were firstly explored, and expected response values were then set based on the quantitative relationships and actual production demands in order to optimize the formula. Simplex-lattice method has been successfully used to optimize the formulas of Waffle ice cream cones (Mahulkar et al., 2024) and novel adsorbent materials (Ghorbani et al., 2021) and the concentrations of O_2_ and CO_2_ for the purposes of maintaining the quality of mango fruits and extending the shelf life (Ntsoane et al., 2020). In recent years, the method has also been well applied in edible fungus cultivation. With the method, Wu et al. (2019a) optimized a high-yield formula of *Pleurotus pulmonarius*, which increased biological efficiency by 15.2% and shortened the fertility period by 6 days. Song et al. (2018) used the method to optimize a high-yield formula of *Grifola frondosa*, which increased the yield by 39.97% and the biological efficiency by 38.53% compared to its control formula. In this study, we also optimized a formula with wood replaced by grass for the mycelial growth of *H. erinaceus* by setting the expected response value of each evaluation index according to the major crop straw resources in Jilin Province. The biological efficiency of this optimized formula (89.14%) was much higher than that (69.77%) of the wheat straw-based formula (Jahedi et al., 2024). The fertility period of the first mushroom crop was shortened by 7 to 9 days, and even 1 to 2 days shorter than the results of Atila (2019). Its average fresh biomass was also higher than that cultivated from rice straw by Bunroj et al. (2017). In short, the optimized formula could be used as an advantageous formula for *H. erinaceus* cultivation.

The quantitative relationship between each main material and evaluation indexes is the key to further optimize the cultivation formula. In the designed formulas of five kinds of agricultural straw selected in this study, it was found that the interactions between different kinds of straw affected the evaluation indexes such as mycelial growth rate, laccase activity, cellulase activity, and neutral xylanase activity. The interactions between two kinds of straw (soybean straw-rapeseed straw; peanut straw-rapeseed straw) positively contributed to the mycelial growth of *H. erinaceus* and the above combinations of two kinds of straw could maximize the contribution. With the same method, Zhang (2023) studied sand-washing residual mud-based low-carbon gelling materials and also found the interaction between various factors. Wu et al. (2019b) also obtained the same results in the study on the formula of *Pleurotus djamor*. The positive interaction between different kinds of straw might be interpreted as follows. The straw combination provided a more suitable C/N ratio and physicochemical conditions for the mycelial growth of edible fungi (Feng, 2011). Carbon and nitrogen sources are important factors affecting lignin degradation and production of extracellular enzymes by fungi. When various media with different C/N were used to cultivate *Pleurotus geesterani*, the activities of extracellular enzymes, such as laccase and hemicellulase showed significant differences. The limited supply of carbon and nitrogen nutrients could stimulate the fungus to synthesize lignin-degrading enzymes. The mixture of carbon sources showed the more significant stimulation action than a single carbon source (Wang, 2019).

In mushroom formulas studies, the quality and nutrient contents of fruiting bodies were also important agronomic traits for evaluate formulas. Cultivation substrates affect the nutrient composition and nutrient contents of mushroom (Bhattacharjya et al., 2015; Meng et al., 2019; Elkanah et al., 2022). In our study, we found that the contents of K, P and other trace elements in the fruiting bodies of optimized formulas were significantly improved because *H. erinaceus* might better absorb trace elements from cob and rice straw and convert them into own nutritional components. Edible fungi have always been an important source of high-quality proteins (Zied et al., 2017; Yao et al., 2019) and the protein content in *H. erinaceus* is even higher than that in most edible fungi (Zhang et al., 2024). Atila et al. (2021) found that the protein contents of *H. erinaceus* are greatly affected by fruiting temperature. Each strain has the ability to produce fruiting bodies at different temperatures (15 °C, 20 °C, and 25 °C), but the protein content varies greatly. Among them, the protein content of strain He Ankara reached a maximum of 19.7% at 20 °C. Jahedi et al. (2024) cultivated *H. erinaceus* with different crop formulas, and found that carbon nitrogen ratio (C/N) was an important factor affecting the protein content of fruiting bodies. When the nitrogen content in the formula is high, the protein content of the fruiting body is also high (up to 19.33%). In the fruiting bodies obtained with the optimized straw cultivation formula in this study, the content of crude proteins was 152.02 g/kg (15.2%), significantly higher than that of the conventional wood chip formula. The differences between the results of Atila and Jahedi may be caused by differences in strains, fruiting temperature, and the carbon nitrogen ratio of formulas. In the future, we can achieve high protein targeted improvement of *H. erinaceus* 20190111 by adjusting the temperature and formula carbon nitrogen ratio, providing high-quality strain for the *H. erinaceus* industry.

## 5 Conclusion

In this study, a straw cultivation formula was optimized with Simplex-lattice method: 16.3% rice straw, 59.7% cob, 20.0% wheat bran, 2.0% gypsum, 1.0% sucrose, and 1.0% calcium superphosphate. We also found that the mixtures of different kinds of straw as the main material would produce the interactions and affect the mycelial growth rate, laccase activity, cellulase activity, and neutral xylanase activity. In the mushroom production validation experiments, the biological efficiency of the optimized formula was as high as 89% and the fertility period of the first crop of mushrooms was shortened by 7 - 9 days. In addition, the contents of crude proteins and crude fats were also significantly increased. The optimized formula can be used in the production of *H. erinaceus*. This study lays the foundation for expanded cultivation and targeted breeding of varieties of *H. erinaceus*, and is conducive to the rapid development of *H. erinaceus* industry.

## Data Availability

The original contributions presented in the study are included in the article/supplementary material. Further inquiries can be directed to the corresponding authors.
